# Detection of Glycomic Alterations Induced by Overexpression of P-Glycoprotein on the Surfaces of L1210 Cells Using Sialic Acid Binding Lectins

**DOI:** 10.3390/ijms131115177

**Published:** 2012-11-16

**Authors:** Tatiana Bubencíkova, Dana Cholujová, Lucia Messingerová, Danica Mislovicova, Mario Seres, Albert Breier, Zdena Sulova

**Affiliations:** 1 Institute of Molecular Physiology and Genetics, Center of Excellence of the Slovak Research and Development Agency “BIOMEMBRANES2008”, Slovak Academy of Sciences, Vlarska 5, Bratislava 83334, Slovakia; E-Mails: tatiana.kurucova@savba.sk (T.B.); lucia.messingerova@savba.sk (L.M.); mario.seres@savba.sk (M.S.); 2 Cancer Research Institute, Slovak Academy of Sciences, Vlarska 7, Bratislava 83391, Slovakia; E-Mail:dana.cholujova @savba.sk; 3 Institute of Chemistry, Slovak Academy of Sciences, Dubravska cesta 9, Bratislava 84538, Slovakia; E-Mail: chemmisl@savba.sk

**Keywords:** L1210 cells, P-glycoprotein, cell surface sugars, *Sambucus nigra* agglutinin, wheat germ agglutinin, *Maackia amurensis* agglutinin, sialic acid, vincristine

## Abstract

P-glycoprotein (P-gp) overexpression is the most frequently observed cause of multidrug resistance in neoplastic cells. In our experiments, P-gp was expressed in L1210 mice leukemia cells (S cells) by selection with vincristine (R cells) or transfection with the gene encoding human P-gp (T cells). Remodeling of cell surface sugars is associated with P-gp expression in L1210 cells as a secondary cellular response. In this study, we monitored the alteration of cell surface saccharides by *Sambucus nigra* agglutinin (SNA), wheat germ agglutinin (WGA) and *Maackia amurensis* agglutinin (MAA). Sialic acid is predominantly linked to the surface of S, R and T cells via α-2,6 branched sugars that tightly bind SNA. The presence of sialic acid linked to the cell surface via α-2,3 branched sugars was negligible, and the binding of MAA (recognizing this branch) was much less pronounced than SNA. WGA induced greater cell death than SNA, which was bound to the cell surface and agglutinated all three L1210 cell-variants more effectively than WGA. Thus, the ability of lectins to induce cell death did not correlate with their binding efficiency and agglutination potency. Compared to S cells, P-gp positive R and T cells contain a higher amount of *N*-acetyl-glucosamine on their cell surface, which is associated with improved WGA binding. Both P-gp positive variants of L1210 cells are strongly resistant to vincristine as P-gp prototypical drug. This resistance could not be altered by liberalization of terminal sialyl residues from the cell surface by sialidase.

## 1. Introduction

Multidrug resistance (MDR) of neoplastic cells represents an obstacle in the effective treatment of cancer with chemotherapy [1]. Overexpression of the plasma membrane ABCB1 transporter, P-glycoprotein (P-gp), is generally accepted as the most frequent molecular cause for the development of MDR [2]. P-gp overexpression is modulated by nuclear receptors that respond to the presence of xenobiotics as ligands in intracellular space (reviewed in [3,4]). While the activation of pregnane X and constitutive androstane receptors have been described to play a role in P-gp transcription control [5,6], we described retinoic acid receptors that may also play a partial role in this process [4,7]. P-glycoprotein (P-gp) is synthesized as a 145 kDa polypeptide that is glycosylated to a final molecular weight of approximately 175 kDa [8,9]. Massive expression of P-glycoprotein in the plasma membrane leads to the exposure of additive P-gp-linked glycosides, which alters the composition of cell surface sugars. Inhibition of P-gp *N*-glycosylation by tunicamycin leads to increased ubiquitination and the subsequent degradation of P-gp in several P-gp positive cell lines derived from solid tumors via drug selection or transfection with the gene encoding P-gp [10]. However, tunicamycin may also induce P-gp expression at both the mRNA and protein levels that induces increased drug efflux activity in Fao hepatoma cells [11]. We observed total depression of P-gp glycosylation induced by tunicamycin in two P-gp positive L1210 cell variants obtained either by selection with vincristine and or by transfection with the human gene encoding P-gp [12]. Nevertheless, unglycosylated P-gp molecules were still present in the plasma membrane of treated cells, and their drug transport activity remained unchanged. Specific glycosylation of P-glycoprotein could be detected in the plasma membrane of P-gp positive cells by several lectins, such as *Galanthus nivalis* agglutinin (GNA) and *Sambucus nigra* agglutinin (SNA), using lectin blot procedure [8]. However, after tunicamycin treatment of P-gp positive L1210 cells, unglycosylated P-gp could not be detected by either of these lectins [12].

In addition to the direct addition of P-gp-linked glycosides in cell surface sugars pattern, there are secondary alterations in the cell protein glycosylation pathway that are associated with MDR development [13]. Strong depression of UDP-sugars is associated with decreases in glycogen and glycoprotein contents in P-gp positive L1210 cells [14]. Moreover, the interaction between the plasma membrane of P-gp positive L1210 cells with a cationic dye, ruthenium red, was much less intense compared to their P-gp negative counterparts. These data indicated that negatively charged functional groups were depressed on the surface of the plasma membrane of P-gp positive L1210 cells [14]. This negatively charged moiety is believed to be formed predominately by sialic acid (SA) on the cell surface [15,16]. Moreover, a decrease in the number of negative binding sites in the *lamina externa* of resistant cells is not only a sign of an alteration in oligo- and poly-saccharide metabolism but may be related also to changes in cellular aggregation, whereas resistant cells tend to form clusters [17]. This tendency to aggregate may be determined by changes in the number and distribution of negative charges in the glycocalyx or the expression of adhesion molecules [18].

Csuka and Sugar previously described a depression in the agglutination of vincristine-colchicine resistant L1210 leukemic cells by *concanavalin* A (ConA) compared with sensitive L1210 leukemic cells [19]. The alteration of cell surface sugars reduced ConA binding and elevated lectin (*Lycopersicum esculentum* agglutinin, LEA) binding to the cell surface of P-gp positive L1210 cells compared with their P-gp negative counterparts [20]. However, both of these lectins failed to bind to saccharide parts directly linked to P-gp, suggesting considerable changes in the glycoside parts of glycoproteins that are distinct from P-gp in P-gp positive L1210 cells. A similar depression of ConA binding was observed following P-gp overexpression by selection of L1210 cells with vincristine or by transfection of L1210 cells with the human gene encoding P-gp [21]. Taken together, these data indicate that overexpression of P-gp in L1210 cells is directly associated with the physico-chemical alteration of the cell surface due to remodeling of the glycoside parts of several proteins in plasma membrane. These changes include differences in the exposure of negatively charged functional groups (probably SA) on the plasma membrane. Therefore, we sought to study of the interaction between the cell surface of P-gp negative and P-gp positive cells with SNA, lectin from *Triticum vulgaris* (wheat germ agglutinin–WGA) and *Maackia amurensis* agglutinin (MAA), all of which are known as SA-specific lectins [22]. In the present study, we used two variants of L1210 cells that highly express P-gp. These variants were obtained from parental cells (S) via stepwise adaptation to the vincristine (R) [23] or stable transfection with the human gene encoding P-gp (T) [21].

## 2. Results and Discussion

### 2.1. Binding of WGA, MAA and SNA to Glycoproteins in the Crude Membrane Fraction of S, R and T cells

**Figure 1:**
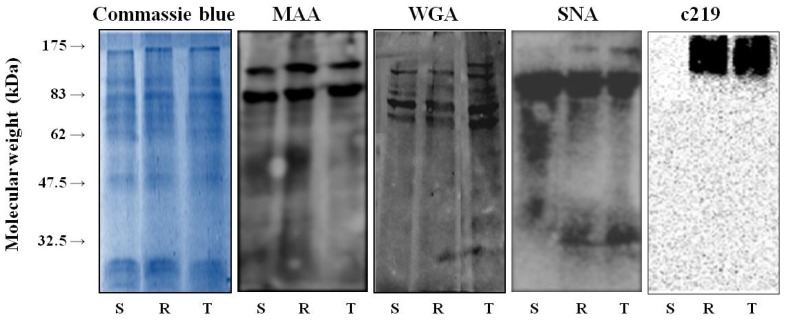
Detection of protein bands in the crude membrane fraction of S, R and T cells by Commassie blue staining of polyacrylamide gels. Wheat germ agglutinin (WGA), *Maackia amurensis* agglutinin (MAA) and *Sambucus nigra* agglutinin (SNA) were detected using lectin blots and western blotting with the c219 anti-P-gp antibody. Polyacrylamide gels were stained with Commassie blue as a control for the accuracy of protein loading. These data are representative of three independent experiments.

Both of the P-gp positive L1210 cell variants, R and T, contained a massive amount of P-glycoprotein that could be detected by western blotting using an anti-P-gp antibody, c219 (Figure 1). In contrast, P-gp was not detectable in crude membrane fractions isolated from S cells. Similarly, both P-gp-positive L1210 cell variants expressed large amounts of P-gp mRNA, whereas this transcript was barely detectable [24] or missing [12,21,25] in S cells. Both P-gp positive variants of L1210 cells (R and T) are strongly resistant to vincristine as prototypical P-gp substrate (Figure S1). SNA detected an approximately 170-kDa band in crude membrane fractions isolated from R and T cells. The corresponding signal was not visible in crude membrane fractions isolated from S cells (Figure 1). These data suggest that SNA binds directly to the saccharide parts of P-glycoprotein. We previously reported a similar direct interaction between P-gp and another lectin, GNA [12]. Recently, P-gp has also been shown to directly interact with SNA and GNA [8]. No protein bands were observed at the 170-kDa region in the presence of WGA and MAA in R and T cells (Figure 1). Similarly to WGA and MAA, also Con A and LEA failed to bind the glycosides linked to P-gp in our recent experiments [20,21]. In contrast, LEA was described to interact with the 180 kDa glycoform of P-glycoprotein in rat brain capillary endothelia and MDR tumor cells [26,27]. This contrast may be explained by different glycosylation patterns of P-gp specific for different cell lines. There are more than 50 P-gp isoforms, which could present a range of differences within the glycome [8].

Changes in glycosylation of other proteins than P-gp may take place in P-gp positive L1210 cell variants because lectin blots of proteins in crude membrane fractions isolated from S, R and T cells with WGA, MAA and SNA revealed an alteration in the glycoprotein profiles between these three L1210 cell variants.

### 2.2. Agglutinations and Cell death Effects of WGA, MAA and SNA on S, R and T Cells

**Figure 2:**
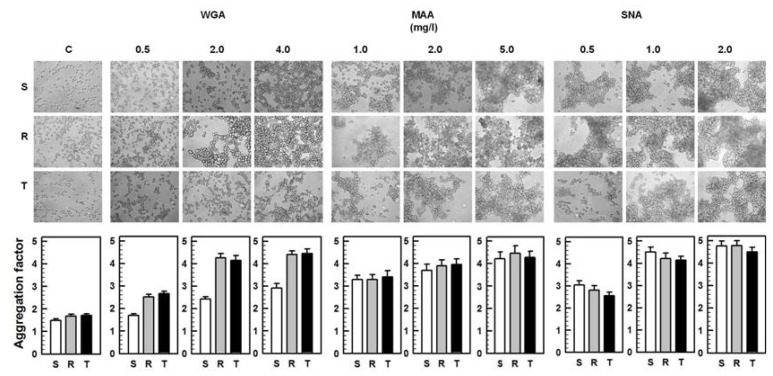
Monitoring of S, R and T cell agglutination by WGA, MAA and SNA. After incubation with lectins (see materials and methods), cells were studied by light microscopy and counted using the CASY Model TT Cell Counter. Microscopic images are representative of three independent experiments. Data are presented as the mean ± SD of three independent experiments.

The agglutination efficiency in S, R and T cells by WGA, MAA and SNA were assessed by light microscopy and quantified as an aggregation factor ascertained using the CASY Model TT Cell Counter (see Materials and Methods). In the absence of lectins, S cells consist predominantly as single cells without the formation of cell aggregates (Figure 2). In contrast, R and T cells tended to cluster under similar conditions. A slight but marginally significant elevation in the aggregation factor was observed for both P-gp positive L1210 cell variants (Figure 2, *p* < 0.1; R and T cells compared with S cells). We previously described a similar tendency for cell clustering in P-gp positive L1210 cell variants [17]. All three of these lectins agglutinated S, R and T cells in a concentration-dependent manner (Figure 2).

WGA agglutinated S cells to a lower extent compared to R and T cells. This behavior was documented by aggregation factors (Figure 2), which were significantly higher for R and T cells compared to S cells at all of the WGA concentration tested (the respective p values were between 0.001 < *p* < 0.02). Similarly to WGA, LEA also agglutinated P-gp positive L1210 cells more potently than their P-gp sensitive counterparts [20]. In contrast, drug sensitive L1210 cells were agglutinated with ConA to a higher extent than their drug resistant variants [19,20]. No significant differences were observed in the ability of WGA to agglutinate R and T cells. In contrast to WGA, MAA agglutinated S, R and T cells with similar efficiency, and no significant changes in the aggregation factors were observed (Figure 2). SNA agglutinated all three variants of L1210 cells more potently than WGA and MAA, as indicated by the lower concentration of lectin necessary for massive cell aggregation (Figure 2).

SNA showed a slightly higher ability to agglutinate S cells than R and T cells, as shown by the lower aggregation factors obtained for R and T cell agglutination compared with S cell agglutination. These differences were marginally significant with probability value between 0.05 < *p* < 0.1.

**Figure 3:**
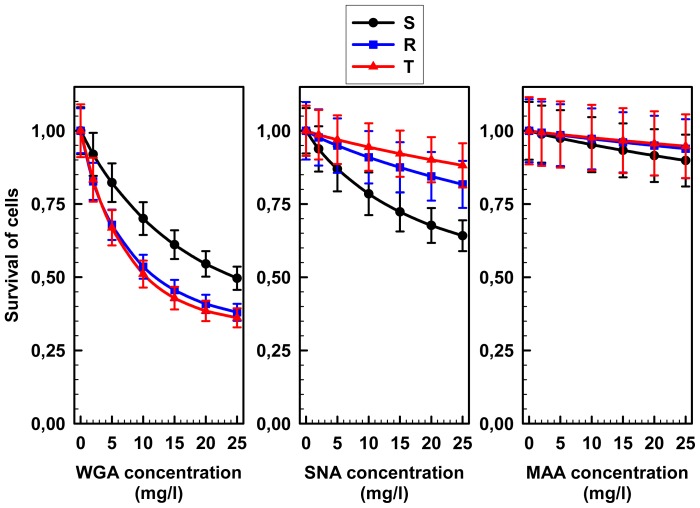
WGA, MAA and SNA induced cell death in S, R and T cells. Cell death was assessed after incubation with lectins (see materials and methods) by spectrophotometric MTT test. Control experiment in the absence of lectins was arbitrarily chosen as 1. Data represent mean ± SD from three independent experiments. Cell death effects induced by WGA and SNA in S cells *vs.* R or T cells were significantly different (0.002 < *p* < 0.02) for all concentrations tested. In R and T cells, differences in cell death in response to these two lectins were not significant. No significant effects on cell death were observed with MAA in all three L1210 cell variants.

WGA exerted cytotoxic effects, to different extents depending on the cell line tested, in toxicity assays [28]. In this study, WGA induced the most pronounced cell death of all three lectins tested (Figure 3). However, SNA was found to agglutinate all three L1210 cell variants more effectively than WGA (Figure 2). Thus, cell aggregation due to agglutination by lectins is not a prevalent inducer of cell death effects in response to these two lectins. We previously made a similar assumption for the interaction between ConA and LEA with the cell surface of P-gp positive and negative L1210 cells [20]. Thus, the cell death effects of several lectins involve specific interaction with several important glycoside parts of glycoproteins integrated in the plasma membrane, which damages crucial cell functions. Recently publish data regarding the cell death effect of lectin ArtinM on human myeloid leukemia cells is consistent with this assumption [29]. P-gp negative S cells were less sensitive to WGA compared to the both P-gp positive cell variants (Figure 3). In contrast, SNA caused greater injury to S cells than to R and T cells. Only weak cell death effects were induced by MAA on all three of the L1220 cell variants (Figure 3).

### 2.3. Binding of Fluorescein Isothiocyanate (FITC) Labeled WGA, MAA and SNA to the Cell Surface of S, R and T Cells

**Figure 4:**
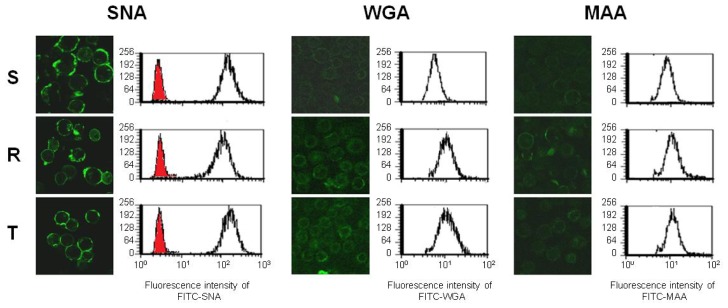
SNA, WGA and MAA binding to the cell surface of S, R and T cells. Cells were studied by confocal microscopy after incubation with Fluorescein Isothiocyanate (FITC) linked lectins (see materials and methods) and were quantified using FACS. The red histograms represent controls in which cells were incubated in the absence of FITC labeled lectins prior to measurement. These data are representative of three independent measurements.

FITC-linked lectins form a compact layer on the surface of L1210 cell variants that are visible by confocal microscopy (Figure 4). We observed similar labeling for FITC-ConA and FITC-LEA attached to the surface of P-gp negative and P-gp positive variants of L1210 cells [20,21]. These data reveal that the indicated FITC linked lectins were not able to enter these cells under these experimental conditions because they were predominantly localized on the cell surface and not in the intracellular space (Figure 4). Cell surface binding of different lectins has also been described for several other cells types [30–34]. Binding of FITC-labeled SNA was very intense to the cell surface of all three L1210 cell variants (Figure 4). The binding of FITC-labeled WGA and MAA were much less pronounced compared to SNA. Furthermore, the binding of SNA was slightly higher on S cells than R and T cells, but this difference was not significant. Alternatively, more intense WGA labeling was observed on both of the P-gp positive L1210 cell variants (R and T) compared to S cells as determined by confocal microscopy and FACS measurements (Figure 4). When the median fluorescent intensities were calculated from FACS histograms, Student’s *t*-test revealed a significant difference between WGA binding onto P-gp negative S cells and P-gp positive R and T cells (*p* < 0.05). Similarly, MAA interacted more intensely with the cell surface saccharides of R and T cells compared to S cells. However, this difference was only marginally significant (*p* < 0.1, using Student’s *t*-test).

### 2.4. Effect of Sialidase Treatment on WGA, SNA and MAA Binding to the Cell Surface of S, R and T Cells

**Figure 5:**
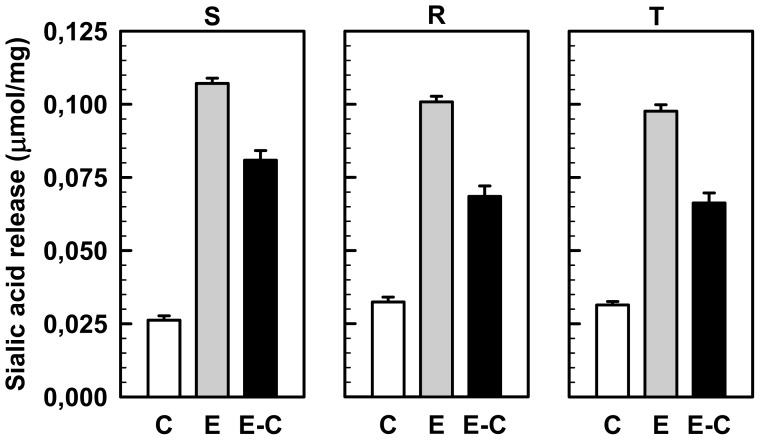
Sialidase induced liberation of sialic acid from S, R and T cells to the external medium. Sialic acid contents were quantified in the external medium after the incubation of cells with sialidase in phosphate buffered saline (PBS) for 10 h (E). As a control, external medium was added after incubation of the cells in the absence of sialidase (C). The net amount of sialic acid liberated by the sialidase reaction represents the difference (E-C). Data represent the mean ± SD of three independent experiments.

All three lectin used in this paper are known to interact with sialyl-glycosides that are present on the cell glycocalyx [22]. SA represents an important component of the cell surface negatively charged moieties that play an important role in cell adhesion and communication [35,36]. Overexpression of P-gp in L1210 cells resulted in the depression of negatively charged targets of the polycation dye, ruthenium red, on cell surface [14,17]. Ruthenium red has been proven to bind directly to cell surface sialyl residues [15,16,37]. Therefore, we chose to study the effects of removing the terminal sialyl groups with sialidase (from *V. cholerae*) on the binding of WGA, MAA and SNA to the cell surface of all three L1210 cell variants. Treatment of S, R and T cells with sialidase liberated SA from the cell surface to the external medium (Figure 5). Treatment of S, R and T cells with sialidase did not alter the response of cells to vincristine (Figure S2), *i.e.*, R and T cells remained much less sensitive to this drug than S cells.

**Figure 6:**
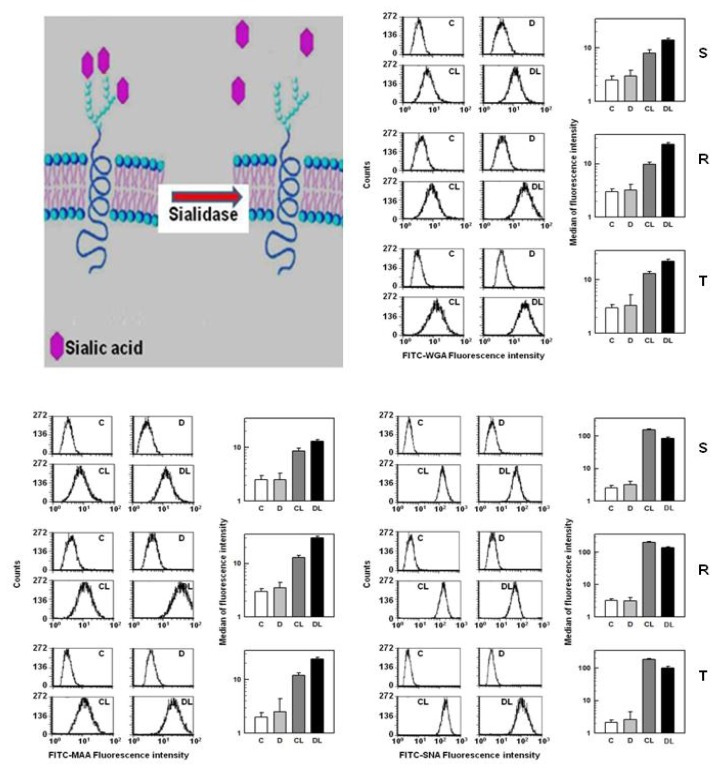
Effects of sialidase treatment on WGA, MAA and SNA binding to S, R and T cells. Sialidase-induced removal of external sialic acid is schematically shown in the upper left panel. Cells incubated in the absence (C) or presence (D) of sialidase were left to interact with FITC-WGA, FITC-MAA and FITC-SNA (CL and DL). Cells incubated in the absence of FITC labeled lectins (C, D) were used as control. FACS histograms are representative of three independent measurements. The median fluorescence intensity data represent the mean ± SD of three independent experiments. Differences between the median fluorescent intensity were significant when comparing CL and DL with the following probabilities for WGA binding to: S cells—*p* < 0.02, R cells—*p* < 0.01 and T cells—*p* <0.01; MAA binding to S cells—*p* < 0.02, R cells—*p* < 0.01 and T cells— *p*< 0.01; and SNA binding to S cells—*p* < 0.005, R cells—*p* < 0.01 and T cells—*p* < 0.01.

Bacterial sialidases (EC 3.2.1.18) hydrolyze the α-2,3, α-2,6 and α-2,8 branched terminal SA residues from oligosaccharides, glycoproteins and glycolipids (Figure 6) [38]. The net quantity of SA liberated following the incubation of R and T cells with sialidase was lower than that of S cells. These differences were found to be marginally significant (*p* < 0.1), indicating that a slightly lower number of terminal sialic residues were accessible for sialidase on R and T cells compared to S cells. Positive signals observed with SA in control experiments (*i.e.*, after incubation of these cells in the absence of sialidase) were originated from materials (glycolipids, glycopeptides or oligosaccharides) released from cells to the external medium during the incubation of cells under non-growth condition in PBS.

This release was more pronounced in P-gp positive R and T cells than in P-gp negative S cells, indicative of P-gp export activity. However, P-gp positive L1210 cells also exported metalloproteinases, important extracellular enzymes, to higher extent than their P-gp sensitive counterparts [39]. The transmembrane efflux of metalloproteinases cannot be attributed to P-gp function, therefore the extrusion of intracellular materials to the extracellular medium, independent of P-gp efflux activity, should also be considered in this process.

The removal of external SA improved the binding of FITC-WGA and FITC-MAA to the external surface of all three L1210 cell variants (Figure 6). In contrast, FITC-SNA was labeled cells after sialidase treatment to lower extend.

All of the three lectins used in the current study are known as SA specific lectins [22]. However, they differ in the terminal SA linkage to the oligosaccharide branch. The specificities of these lectins are documented in Table 1.

The binding of SNA to the cell surface of S, R and T cells was found to be higher than other two lectins. (Figures 4 and 6). This effect was associated primarily with amplified cell agglutination for SNA in comparison to MAA and WGA (Figure 2).

These results indicate that a majority of SA is linked to S, R and T cell surfaces via the α-2,6 branches that recognizes SNA and not via the α-2,3 branches that recognizes MAA. The removal of terminal SA by sialidase damages SNA ligands on the cell surface and therefore depressed SNA binding to all L1210 cell variants. In contrast to SNA, the removal of SA from cell surface of S, R and T cells by sialidase improved MAA binding, a result that seems to be controversial. This observation could be explained by a report by Knibbs *et al.*[41], which demonstrated that SNA is required for the binding of disaccharides with the following structures: SA-5-acetyl-α-2,6-galactose or SA-5-acetyl-α-2,6-*N*-acetyl-galactosamine. These researchers also reported that MAA has a binding site complimentary to the trisaccharides SA-5-acetyl-α-2,3-galactose-1,4-*N*-acetyl-glussamine or SA-5-acetyl-α-2,3-galactose-1,4-glucose, to which sialic acid contributes less to the total binding affinity than SNA. Thus, if SA is linked to the surface of S, R and T cells via α-2,6 branches, this ligand could not be appropriate for MAA. However, removing sialic acid from this branching with sialidase may improve the exposure of internal saccharide structures (with MAA binding characteristics) on the surfaces of S and R and T cells and will consequently elevate MAA binding. The fact that SNA seems to interact (Figure 4), agglutinated (Figure 2) and damaged (Figure 3), more potently with S cells than R and T cells indicates that S cells contain a slightly higher amount of α-2,6 branched SA than R and T cells. In contrast, MAA interacts more effectively with the surface of R and T cells compared to S cells in sialidase treated or untreated cells (Figure 6), indicating that the internal saccharide structures that are ligands for MAA are exposed more effectively in P-gp positive cells (R and T) compared to P-gp negative (S) cells. WGA recognizes internal *N*-acetyl-glucosamine and bound less effectively to SA linked to *N*-acetyl-glucosamine and other structures (Table 1). Removal of SA from the cell surface by sialidase treatment opens the structure of external cell glycocalyx and will increase the accessibility of *N*-acetyl-glucosamine and other WGA ligands to WGA, which will elevate WGA binding. Elevated binding of WGA was proven by the data in Figure 6. Structures that are ligands for WGA were found to be more accessible for WGA in P-gp positive R and T cells compared to P-gp negative S cells in either untreated or sialidase-treated cells. These structures seem to contain *N*-acetyl-glucosamine, and therefore this amino-sugar must be present in higher amounts on P-gp positive R and T cells compared to P-gp negative S cells. These data are consistent with more pronounced binding of LEA to P-gp positive than P-gp negative L1210 cell variants, as described in our previous paper [20]. LEA recognizes chitin oligosaccharides that contain clusters of *N*-acetyl-glucosamines [42]. Further studies are necessary to determine if WGA and MAA recognize common ligands on the surface of the L1210 cell variants used in this paper.

Several authors have described any alterations of protein glycosylation status as consequence of treatment with anticancer agents [43,44]. These alterations were suggested to be caused by long term exposure of cancer cells to chemotherapeutic agents as side effect of cell adaptation/selection process. In the contrast, change in composition of cell surface sugars detected by specific lectins in the present paper seems to be directly related to P-gp overexpression and is independent on way by which P-gp expression was achieved. This suggestion could be deduced from fact that R and T cells show similar interaction with all three of the lectins applied in this study, and this behavior differs in S cells. Therefore, differences in SNA, WGA and MAA binding to R and S cells could not be attributed to process of cell adaptation/selection with vincristine.

## 3. Experimental Section

### 3.1. Cell Culture Conditions

The following L1210 cell variants were used in this study: (i) S, drug-sensitive parental cells; (ii) R, P-gp-positive, drug-resistant cells that overexpress P-gp after selection with vincristine (VCR, purchased from Gedeon Richter Co., Hungary) [23]; and (iii) T, P-gp-positive, drug-resistant cells that overexpress P-gp following stable transfection with the P-gp gene [21] using the Addgene plasmid, 10957 (pHaMDRwt), a retrovirus encoding the full-length P-gp cDNA [45]. The cells (S, R and T; inoculums 1 × 10^6^ cells) were cultured in 4 mL RPMI 1640 media with l-glutamine (1 mg/mL), 4% fetal bovine serum and 1 μg/mL gentamycin (all purchased from Gibco, (Langley, OK, USA)) in a humidified atmosphere with 5% CO_2_ and at 37 °C. R cells were cultured for two passages without VCR prior to the experiments.

### 3.2. Western Blot and Lectin Blot Procedures

P-gp and other membrane glycoproteins were detected by western blot and lectin blot procedures in crude membrane fractions isolated from S, R and T cells. Crude membrane fractions were prepared with a ProteomeExtract Subcellular Proteome Extraction Kit (Calbiochem, San Diego, CA, USA). Proteins in the samples were separated by sodium dodecyl sulfate polyacrylamide electrophoresis (SDS-PAGE) on polyacrylamide gradient gels (8–16%). Proteins were then transferred by electroblotting to nitrocellulose membranes (GE Healthcare Europe GmbH, Vienna, Austria). The C219 anti-P-gp monoclonal antibody (Calbiochem, San Diego, CA, USA) was used to detect P-gp by western blot. An anti-mouse secondary antibody conjugated to horseradish peroxidase was used for detection with the aid of the ECL detection system (GE Healthcare Europe GmbH, Vienna, Austria) and a Kodak scanning system CF 440 (New Haven, CT, USA). For glycoprotein detection by lectin blots, WGA, MAA and SNA were conjugated with biotin (EY Laboratories Inc. San Mateo, CA, USA), and avidin was conjugated with horseradish peroxidase (Sigma, St. Louis, MO, USA). The peroxidase signals were detected using the ECL detection system and a Kodak CF 440 scanning system. Commassie blue (Sigma, St. Louis, MO, USA) staining of polyacrylamide gels was used to verify the accuracy of protein loading.

### 3.3. Detection of S, R and T Cells Agglutination by WGA, MAA and SNA

Cells (1 × 10^5^) were incubated with different concentrations of WGA, MAA and SNA (EY Laboratories Inc., USA) in 1 mL of RPMI medium without bovine fetal serum for 2 h in a humidified atmosphere with 5% CO_2_ at 37 °C. Then, the agglutination of cells was monitored under a light microscope and aggregation factor was quantified using a CASY Model TT Cell Counter (Roche Applied Sciences, Indianapolis, IN, USA). The aggregation factor of a sample is automatically calculated as the quotient of the mean and peak cell volumes [46].

### 3.4. Effect of WGA, MAA and SNA on S, R and T Cell Survival

As previously described [20], cells were cultivated in 96-wells plates (5 × 10^4^ cells/well in 200 μL medium) in the absence or presence of different concentrations of WGA, MAA and SNA. After cultivation, cell viability was assessed by MTT test (Thiazolyl Blue Tetrazolium Bromide) [47].

### 3.5. Detection of FITC Labeled WGA, MAA and SNA Binding to the Surface of S, R and T Cells by Confocal Microscopy and Flow Cytometry

After cultivation, cells were washed three times with PBS, resuspended in RPMI medium without fetal bovine serum (5 × 10^5^ cells/mL) and incubated for 60 min with FITC-labeled WGA, MAA and SNA (EY Laboratories Inc. San Mateo, CA, USA) at a concentration of 1 mg/L in a humidified atmosphere supplemented with 5% CO_2_ at 37 °C [20]. After incubation, the cells were washed three times with PBS, and specific labels were evaluated by green fluorescence with a confocal laser-scanning microscope (LSM 510 META, Carl Zeiss) or were counted with a BD Accuri C6 flow cytometer (BD Bioscience, San Jose, CA, USA). In a special set of experiments, cells that showed prior interaction with FITC labeled lectins were treated with sialidase.

### 3.6. Treatment of S, R and T cells with Sialidase and Estimation of the Amount of Sialic Acid Released from Cells to the External Medium

Cells (1 × 10^7^) were treated with sialidase from *V. cholerae* (Roche Applied Sciences, Indianapolis, IN, USA; 0.5 U/mL) in 1 mL of sterile phosphate buffered saline (PBS) containing 1% BSA (Merck Slovensko) and a protease inhibitor cocktail (Roche Applied Sciences, Indianapolis, IN, USA) for 10 hours. Next, cells were separated by centrifugation and used to estimate the binding of FITC-labeled WGA, MAA and SNA by FACS. Cell viability was monitored by propidium iodide staining using a BD Accuri C6 flow cytometer, and only propidium iodide negative cells were included in the FACS histograms. After centrifugation, the supernatants were used to estimate the amount of SA released with a thiobarbituric acid assay for sialic acids according to Warren’s method [48].

### 3.7. Statistical Analysis and Data Processing

Numerical data are expressed as the mean ± SD of three independent measurements. Statistical significance was assessed using an unpaired Student’s *t*-test using SigmaPlot Graphing Software (version 2.01; San Jose, CA, USA).

The concentration dependent cytotoxic effects of WGA, MAA and SNA on cell viability were fitted according to an equation for exponential decay as previously described [21].

## 4. Conclusions

R and T cells show similar interaction with all three of the lectins applied in this study, and this behavior differs in S cells. In this study, we measured cell agglutination, cell death effects induced by lectins and lectin binding to cell surfaces (Figures 2–4 and 6). Thus, alterations in the exposure of specific saccharide ligands for lectins on cell surface of L1210 cells is directly associated with the presence of P-gp in cells and is independent of means by which P-gp expression was achieved. As was previously hypothesized [3,21], this feature is involved in the complex remodeling of cell surface glycosides, which takes place as a secondary cellular response to P-gp expression in plasma membrane of L1210 cells.

Sialic acid is linked to the surface of S, R and T cells, predominantly via α-2,6 branched sugars, resulting in massive binding of SNA. Binding of MAA was much less pronounced, indicating only a minor contribution of α-2,3 branched SA to the total pool of SA located on cell surface of all three L1210 cell variants. P-gp positive R and T cells should contain higher amounts of *N*-acetyl-glucosamine on their cell surface compared to S cells, which is consistent with heightened WGA binding.

The cell death effect of lectins did not correlate with their binding efficiency and agglutination potency. Thus, the more pronounced cell death effect observed with WGA compared to SNA, which is more efficiently bound to the cell surface compared to WGA, indicates that the specific attachment of lectin to sugar parts of glycoprotein depresses its function and is essential for cell survival. A similar assumption can also be made for ConA-induced cell death effects in P-gp negative and positive L1210 cell variants, as described previously [20].

## Supplementary Information



## Figures and Tables

**Table 1:** Ligand specificity of WGA, MAA and SNA.

Lectin from	Abbreviation	Specificity
*Triticum vulgaris* (Wheat germ)	WGA	Internal β-d-*N*-acetyl glucosamine> β-d-*N*-acetyl glucosamine–SA > *N*-acetyl galactosamine > lactose > galalactose
*Maackia amurensis*	MAA	α-2,3 branched SA
*Sambucus nigra*	SNA	α-2,6 branched SA

Data were adopted from [22,40].
