# Supplementary material for: Detection of Glycomic Alterations Induced by Overexpression of P-Glycoprotein on the Surfaces of L1210 Cells Using Sialic Acid Binding Lectins
Source: Int J Mol Sci. 2012 Nov 16;13(11):15177–92. doi: 10.3390/ijms131115177 (PMC3509634; doi:10.3390/ijms131115177)

Supplementary Information

Figure S1. Cytotoxic effects of vincristine on S, R and T cells. Cells death was assessed after cultivation of cells in cultivation medium for two days in the presence or absence of vincristine at respective concentration by spectrophotometric MTT test. Cultivation was carried out at 200 μl of cultivation medium at 96 wells cultivation plates (inoculums 5 × 10^4 cells/well). Data represent the mean±S_d of six independent measurements. R and T cells are strongly resistant to vincristine.

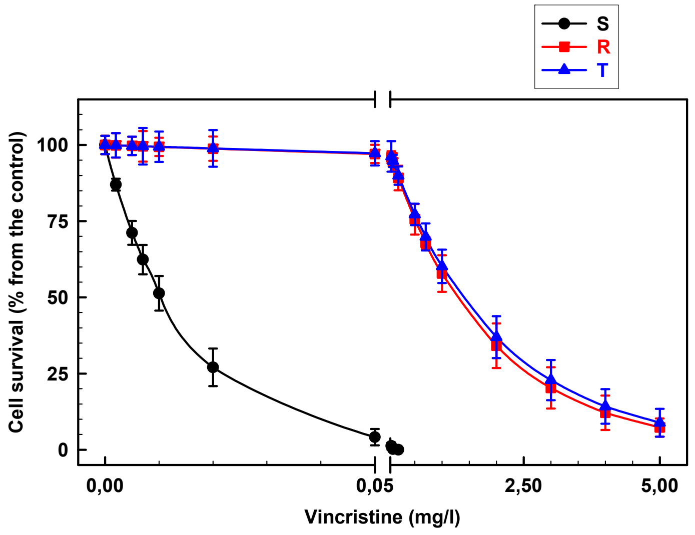

Figure S2. Effect of sialidase treatment on vincristine sensitivity of S, R and T cells. Cells (2 × 10^6) were incubated in 96 wells cultivation plates with sialidase (0.5 U/mL) and vincristine (1 mg/L) in 200 μL of sterile phosphate buffered saline containing 1% BSA (Merck Slovensko) and a protease inhibitor cocktail (Roche Applied Sciences, USA) for 24 hours in a humidified atmosphere with 5% CO_2 and at 37 °C. After incubation viability of cells were estimated by MTT test. White columns – controls (without treatments); red columns – treatment with sialidase; blue columns – treatment with vincristine; black columns – treatments with vincristine and sialidase. Data represent the mean±S_d of six independent measurements. Sialidase treatment did not induce any considerable changes of S, R and T sensitivity to vincristine.

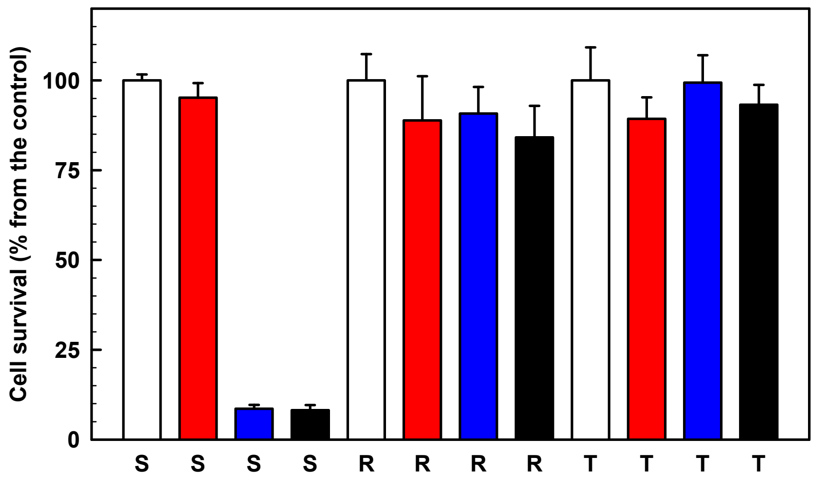

Supplement: Supplementary file 1 [file ijms-13-15177-s001.pdf]
